# Vessel co-option in primary human tumors and metastases: an obstacle to effective anti-angiogenic treatment?

**DOI:** 10.1002/cam4.105

**Published:** 2013-07-08

**Authors:** Tom Donnem, Jiangting Hu, Mary Ferguson, Omanma Adighibe, Cameron Snell, Adrian L Harris, Kevin C Gatter, Francesco Pezzella

**Affiliations:** 1 Department of Oncology, University Hospital of North NorwayTromso, Norway; 2 Institute of Clinical Medicine, University of TromsoTromso, Norway; 3 Nuffield Department of Clinical Laboratory SciencesJohn Radcliffe Hospital, University of OxfordOxford, United Kingdom; 4 Department of OncologyWeatherall Institute of Molecular MedicineJohn Radcliffe Hospital, University of OxfordOxford, United Kingdom

**Keywords:** Angiogenesis, cancer, lung cancer, nonangiogenic tumors, tumor growth, vessel co-option

## Abstract

Angiogenesis has been regarded as essential for tumor growth and progression. Studies of many human tumors, however, suggest that their microcirculation may be provided by nonsprouting vessels and that a variety of tumors can grow and metastasize without angiogenesis. Vessel co-option, where tumor cells migrate along the preexisting vessels of the host organ, is regarded as an alternative tumor blood supply. Vessel co-option may occur in many malignancies, but so far mostly reported in highly vascularized tissues such as brain, lung, and liver. In primary and metastatic lung cancer and liver metastasis from different primary origins, as much as 10–30% of the tumors are reported to use this alternative blood supply. In addition, vessel co-option is introduced as a potential explanation of antiangiogenic drug resistance, although the impact of vessel co-option in this clinical setting is still to be further explored. In this review we discuss tumor vessel co-option with specific examples of vessel co-option in primary and secondary tumors and a consideration of the clinical implications of this alternative tumor blood supply.

Both primary and metastatic tumors use preexisting host tissue vessels as their blood supply. Tumors may grow to a clinically detectable size without angiogenesis and makes them less likely to respond to drugs designed to target the abnormal vasculature produced by angiogenesis, but further studies to explore the biological and clinical implication of these co-opted vessels is needed.

## Introduction

Angiogenesis, the development of new blood vessels following the proliferation of the endothelial cells of preexisting vessels, is regarded as a hallmark in cancer development 1. There is, however, evidence that both primary tumors and metastases are able to progress without angiogenesis. Examples of such tumors include subgroups of non–small cell lung tumors (NSCLC) 2–3, lymphoma 4, and glioblastoma multiforme (GBM) 5. Furthermore, nonangiogenic metastatic tumors have been described in lung 6, liver 7,8, and lymph nodes 10. This challenges the hypothesis that angiogenesis is required for tumor growth and suggests that the vasculature of human tumors is more complicated than initially anticipated.

Several types of nonangiogenic tumor vascularization have been described and defined as seen in Table 1. Vasculogenic mimicry (VM) is a mechanism by which highly aggressive tumor cells can form vessel-like structures themselves, by virtue of their high plasticity. A review by Paulis et al. 11 describes signaling pathways in VM and its role in on-going cancer research. Another mechanism is by intussusception where preexisting vessels split into daughter vessels. In an interesting study by Gianni-Barri et al. 12: “To sprout or to split? VEGF, Notch and vascular morphogenesis,” they explore intussusception as an alternative tumor blood supply and the molecular regulation of this process.

**Table:** Modes of vessel formation in normal and tumor tissue

		Normal tissue	Tumor tissue
Vasculogenesis	In developing mammalian embryo angioblasts differentiate into endothelial cells assembling into vascular labyrinth. Distinct signals differentiate arterial or venous differentiation	V	V
Angiogenesis	Endothelial sprouting, the development of new blood vessels following the proliferation of the endothelial cells of preexisting vessels	V	V
Arteriogenesis	Endothelial cell channels become covered by pericytes or vascular smooth muscle cells (VCAMs)	V	V
Intussusception	Preexisting vessels split into daughter vessels	V	V
Vessel co-option	Tumor cells hijack the existing vasculature. Tumor cell migration along the vessels of the host organ	–	V
Vascular mimicry	Tumor cells form tubular structures themselves	–	V
Cancer stem-like cells differentiate into ECs	Endothelial cells (ECs) derived from putative cancer stem cells	–	V

Vessel co-option (or vascular co-option) is a mechanism in which tumors obtain a blood supply by hijacking the existing vasculature and tumor cells migrate along the vessels of the host organ. In this review we discuss possible mechanisms for vessel co-option in animal models and describe examples of clinically detectable tumors perfused by vessel co-option. In addition to being important in tumor progression, vessel co-option has been proposed as a potential explanation of why antiangiogenic treatment has less of an effect than expected 13. In a clinical setting, a predictive biomarker for antiangiogenic therapy is not established. Therefore, this issue is likely to be of particular interest to investigators developing clinical trials of drugs that target angiogenesis and for the development of new approaches to the study of tumor angiogenesis. Reliable identification of these tumors is essential for the accurate assessment of antiangiogenic drugs in the most suitable tumors: The review will also describe the morphological growth patterns (GPs) and vascular phenotypes associated with vessel co-option together with clinical implications.

The keywords vessel co-option, nonangiogenic tumors, and angiogenesis were used in a systematic PubMed search and articles judged most relevant were reviewed and included in this report. Table 2 shows original studies published in the last 10 years related to vessel co-option found by the search criteria, and as indicated, much of the recent research related to tumor vessel co-option has used murine or cell line models. However, there are some studies based on human tissue, but compared to the high number of studies related to angiogenesis the focus on vessel co-option as an alternative tumor blood supply has been rather limited. Although potential vessel co-option is observed in many malignancies (Table 2), not surprisingly, vessel co-option seems to be most frequently observed in highly vascularized tissue such as brain, lung, and liver, the former two being well oxygenated, the latter with high nutrient load.

**Table:** Original articles from a systematic search regarded as relevant to describe vessel co-option as an alternative tumor blood supply are shown

Reference	Year	Search	Malignancy	Human	Murine	Cell lines
Van den Eynden et al. 8	2012	2	Liver metastasis from colorectal cancer (CRC)	X		
Budde et al. 26	2012	1	Brain and bone metastasis from breast cancer		X	
Franco et al. 18	2012	1	Pancreatic neuroendocrine tumors (PNAS)		X	
Budde et al. 28	2011	1	Brain metastasis from breast cancer		X	
Zhao et al. 17	2011	1	Malignant melanoma, breast, and colon cancer			X
Di Tomaso et al. 31	2011	1	Glioblastoma	X		
Auf et al. 20	2010	1	Glioma		X	X
Kienast et al. 27	2010	1	Brain metastasis from lung cancer and melanoma cell lines		X	
Helfrich et al. 19	2010	3	Melanoma	X	X	
Carbonell et al. 49	2009	1	Brain metastasis from different malignancies	X	X	X
Winkler et al. 50	2009	1	Glioma		X	
Reiss et al. 51	2009	1	Breast cancer		X	
Sardari et al. 24	2008	3	Non–small cell lung cancer (NSCLC)	X		
Sardari et al. 6	2007	2	Lung metastasis from renal cell carcinoma	X		
Offersen et al. 52	2007	2	NSCLC	X		
Adighibe et al. 21	2006	2	NSCLC	X		
Arismendi-Morillo et al. 53	2005	1	Brain	X		
Renyi-Vamos et al. 54	2005	2	NSCLC	X		
Hu et al. 29	2005	2	NSCLC	X		
Paku et al. 55	2005	3	Liver metastasis		X	X
Leenders et al. 38	2004	1	Brain metastasis from malignant melanoma cells		X	X
Shieh et al. 56	2004	2	Oral squamous cell carcinoma	X		
Sardari et al. 57	2004	2	NSCLC	X		
Stessels et al. 7	2004	2	Liver metastasis from CRC and breast cancer	X		
Guedj et al. 58	2004	3	Lung cancer (bronchoalveolar carcinoma, BAC)	X		
Kaicker et al. 59	2003	1	Neuroblastoma		X	
Leenders et al. 60	2003	1	Brain metastasis from malignant melanoma		X	
Passalidou et al. 4	2003	3	Non-Hodgkins lymphoma	X		

Human-, murine-, and cell line studies are included. Studies published last 10 years are shown.

Search criteria – systematic search in PubMed January, 2013. Search 1: “vessel co-option” OR “vessel cooption” OR “vessel co option” AND “cancer” – 370 hits, 20 regarded relevant, 14 last 10 years. Search 2: “non-angiogenic” OR “nonangiogenic” – 126 hits, 13 regarded as relevant, 9 last 10 years. Search 3: Cited in studies from Searches 1 and 2, 15 regarded relevant, 5 last 10 years.

## Animal Models of Tumor Vessel Co-Option

Early observations in human brain tissue that demonstrated how a subset of tumors initially grow by co-opting existing host vessels inspired Holash et al. to do further studies in murine models 5–14. Holash and coworkers observed the process of vessel co-option to be followed by vessel regression, tumor hypoxia, and the stimulation of angiogenesis for further growth to be based on the relative expression of pro- and antiangiogenic endothelial growth factors (angiopoietin-1 and -2 and vascular endothelial growth factor, VEGF) 14. Interestingly, in their rat glioma model, the co-opted vessels of the early tumors expressed high levels of angiopoietin-2, the natural antagonist to the angiogenic angiopoietin-1. As the tumors grew and became increasingly hypoxic, VEGF expression was seen at the hypoxic periphery of the larger tumors. The process of vessel co-option was not limited to gliomas, as shown by using rat mammary adenocarcinoma in the same model conditions. The adenocarcinomas rapidly co-opted blood vessels. It might be argued that the specialized conditions in the brain do not accurately model conditions elsewhere: The authors, however, injected Lewis Lung Carcinoma cells intravenously to colonize the lung with similar results.

The idea of initial vessel co-option was later supported by Kusters et al. 15. In one of several comprehensive murine studies related to this topic done by this group, they induced metastasis to mouse brain parenchyma by injection of melanoma cell lines into the carotid artery. Lesions with diameters up to 3 mm^3^ were formed showing an infiltrative GP in the parenchyma, which exploited preexisting brain vessels. There were no differences between the intratumoral vessels and vessels in normal brain (assessed by vessel diameter, pericyte coverage, and state of endothelial activation) and they had the characteristics of an intact blood–brain barrier and vessel density was slightly lower than in the surrounding normal brain. Interestingly, when the injected melanoma cell lines were engineered to express the potent angiogenic factor VEGF_165_, despite endothelial cells being activated and showing upregulation of kinase insert domain receptor (KDR) and endothelial cells and their surrounding pericytes responding to the VEGF by proliferation, there was no induction of angiogenesis in terms of sprouting and branching of new vessels. Although challenged by other investigators who have used the same rat glioma model 16, Kusters et al. 15 suggest that tumor cells do have the capacity to co-opt vessels, allowing nonangiogenic growth, and the models begin to provide an explanation of the molecular mechanisms behind the process.

More recent studies have also emphasized vessel co-option as an important alternative blood supply and provided further insight to this mechanism's role in tumor development. In a zebrafish study by Zhao et al. 17 it was concluded that the vessel co-option and angiogenesis have distinct contributions at the earliest stage of microtumors initiation and metastasis. However, they suggest that angiogenesis plays the critical role in tumoral exponential growth, whereas the strategy of co-opting host vessels is an alternative but essential choice for tumor cells to survive. Interestingly, they also found some tumor cells in brain co-opted vessels with vessel-like pseudopodia making them cover the vessel surface maximally and obtain more support from the host, such as nutrients and oxygen.

In addition, to further explore the role of vessel co-option in tumor development, recent murine studies have supported the idea that vessel co-option may be a potential explanation as to why antiangiogenic therapy in many cases does not appear to be as beneficial as initially expected. Franco et al. 18, by the use of a genetically engineered mouse model of pancreatic neuroendocrine tumors (PNET), observed that tumors refractory to VEGFR-2 blocking antibody treatment contained blood vessels with a prolific investment of pericytes expressing α-smooth muscle actin (α-SMA). The authors claim it is likely that the blood vessels carrying α-SMA^+^ pericytes present within resistant tumors are derived from co-opted blood vessels. This conclusion is in agreement with a murine melanoma study identifying blood vessels covered by α-SMA^+^ pericytes as a particular feature of tumors acquiring vascularization through a nonangiogenic mechanism 19. In this latter study, they also analyzed human melanoma metastases taken at clinical relapse in patients undergoing adjuvant treatment with bevacizumab, in addition to the murine model in which melanomas developed spontaneously. In both settings, those tumors developing during anti-VEGF therapy were characterized by a mature intratumoral vascular network showing low angiogenic activity and this vascular phenotype was independent of tumor volume or localization. However, there are indications that also newly formed vessels may recruit α-SMA^+^ pericytes. Therefore, SMA^+^ vessels are not necessarily co-opted and SMA positivity is not a confirmed definitive marker for co-opted vessels.

Interestingly, using both the chick chorioallantoic membrane assay and a mouse orthotopic brain model, Auf et al. 20 observed that inhibition of inositol-requiring enzyme 1 (IRE1) correlated with downregulation of proangiogenic factors and upregulation of antiangiogenic gene transcripts. Blockade of IRE1 modified glioma expansion by reducing angiogenesis and by promoting tumor cell invasion. Furthermore, the glioma cells co-opted the host vasculature and infiltrated the brain along blood vessel tracks.

The three latter murine studies have been able to detect vessel co-option as a potential explanation of why antiangiogenic therapy may not work 18,19. However, many traditional tumor xenograft models involve the inoculation of tumor cells in basically avascular subcutaneous space making them induce angiogenesis without the opportunity to use vessel co-option as an alternative blood supply. This is important as many of the promising preclinical studies of antiangiogenic drugs have been done in such relatively avascular experimental tumors.

## Identification of Tumor Vessel Co-Option

Many strategies have been used to identify putatively nonangiogenic tumors including microvessel density (MVD) counting, markers of endothelial cell proliferation, morphology, and new imaging techniques. Unfortunately, distinguishing newly formed (angiogenic) vessels from mature “nonangiogenic” vessels co-opted by a tumor is difficult.

**Figure 1:**
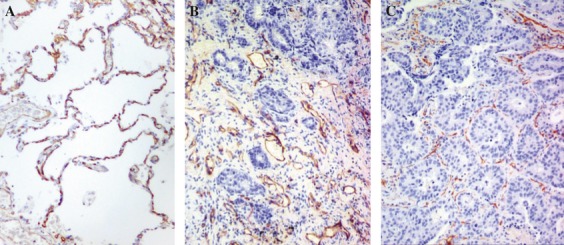
(A) Normal lung. Immunostaining for CD31 (antibody JC70) demonstrates the capillaries (in red) of the normal alveoli. (B) An angiogenic tumor: The normal lung architecture is diffusely replaced. New vessels (in red) and stroma are produced intimately mixed with neoplastic cells but without any recognizable architectural structure. (C) A nonangiogenic tumor. Section of lung in which a carcinoma is growing by filling the alveolar spaces: staining for CD31 shows the co-opted alveolar vessels highlighting the normal lung architecture. In this case the pattern is present throughout the whole lesion.

However, many of the tumors that have been reported to grow without angiogenesis have a distinctive morphology which in many cases allows their identification by light microscopy (Fig. 1). Nonangiogenic tumors of the lung, for example, are characterized by the “chicken-wire” appearance of the preserved alveoli through which they grow 21,22. Complicating the picture, tumors with a mixed phenotype is often seen, suggesting that nonangiogenic tumors, rather than being a distinct subtype of tumor, are probably only one extreme of a fairly widespread process occurring in many tumors particularly at the active edge. In the mixed cases the vessel co-option component often are observed at their actively growing edges, with the more mature center showing a switch to an angiogenic phenotype.

Studies of angiogenesis in human tissues have often used the technique of MVD measurement as a benchmark of angiogenic activity. However, the MVD of a tumor does not necessarily correlate with angiogenic activity in the tumor. A major drawback of the technique is in its failure to take into account the potential presence in a tumor of co-opted, mature vessels. Although tumor MVD counts that are similar to those obtained for the normal surrounding tissue may provide an indirect sign of nonangiogenic growth, these need to be used in combination with other methods of assessing angiogenesis. Several antibodies including CD31, CD34, and vWF (von Willebrand factor)/FVIII (Factor VIII) are useful for staining endothelium, but do not differentiate between mature and immature blood vessels. Mature vessels are characteristically surrounded by pericytes. When stained for smooth muscle actin (SMA) nonangiogenic vessels show greater levels of SMA than their less mature angiogenic counterparts. LH39 is an antibody directed against an epitope of the lamina lucida of the basement membrane and is expressed by capillaries and small venules in normal tissues. A study by Passalidou et al. 22 was carried out to determine phenotype and LH39 was expressed by vessels in normal and nonangiogenic tumors, but on only a small minority of angiogenic tumor vessels.

The fraction of proliferating endothelial cells, highlighted by double immunoreactivity with endothelial cell-specific antibodies such as CD34 or Factor VIII and cycling nuclei-specific antibodies such as Ki67 or proliferating cell nuclear antigen (PCNA) has been proposed as a more reliable measure of on-going angiogenesis than MVD. Sardari et al. 24 used double immunostaining with CD34 and Ki-67 antibodies to assess endothelial cell proliferation fraction and classified NSCLC into GPs based on morphological characteristics of the tumor tissue at the invading front; alveolar nonangiogenic (co-option), papillary intermediate (co-option and angiogenesis), and destructive angiogenic (angiogenesis).

Imaging techniques and functional imaging in clinical trials of targeted therapies were recently reviewed and imaging of angiogenesis and hypoxia was discussed 25. Dynamic contrast-enhanced (DCE), magnetic resonance imaging (MRI) is put forward as a perfusion imagining technique able to exploit differences between “leaky”, disorganized, tumor neovessels, and normal, well-organized vasculature. However, the picture is complex and in a recent study by Budde et al. 26, the authors conclude that DCE-MRI seemed to be a good alternative to evaluate bone metastasis from breast cancer, but less useful monitoring brain metastasis from the same primary tumor. Other imaging methods, however, have also been explored. Kienast et al. 27 have established multiphoton laser scanning microscopy to image single steps of mouse brain metastasis formation in real time. In this model they monitored arrest at vascular branch points, early extravasation, persistent close contacts to microvessels, and, finally, differentiating perivascular growth between vessel co-option and early angiogenesis. Kienast and coworkers observed that brain metastasis initially proliferating by co-opting vessels needed remodeling of the existing vasculature for the continuous growth of micrometastasis. Furthermore, they found that successful brain macrometastasis formation in mice given lung cancer cell lines was followed by angiogenesis. In mice injected with melanoma cells, initially growing micrometastasis that later regressed was located in regions of poor vascularization compared to those micrometastasis that grew to macrometastasis, indicating vessel co-option as pivotal for tumor development in this cells line. Although the technique is intriguing and offers advantages in a mouse model setting, there is a long way to go before it is practical in a clinical setting. In another murine study, Budde et al. 28 conclude that phase contrast MRI is sensitive to vascular remodeling in co-opting brain tumor metastasis independently of sprouting angiogenesis, and may therefore be useful in preclinical studies of angiogenic-independent tumors or in the monitoring of continued tumor growth following antiangiogenic therapy. However, in conclusion, several imaging techniques are in use to evaluate antiangiogenic treatment and to differentiate between angiogenesis and vessel co-option. Both the tumor type and location seem to be an issue and further studies are needed to explore and optimize their clinical impact.

## Metabolism, Inflammation, and Apoptosis in Tumors With Co-Opted Blood Supply

Together with inflammation, energy metabolism has been introduced as a new hallmark of cancer and there is evidence that metabolism is differently expressed in vessel co-opted versus angiogenic tumors 1–29. cDNA microarray analysis has been carried out in our laboratory to compare nonangiogenic with angiogenic lung tumors 29. Tumors with co-opted blood supply had higher levels of genes coding for proteins involved in mitochondrial metabolism. This finding suggests a more effective regulation of the intracellular respiratory chain in these tumors. A possible explanation may be that the oxygen tension near normal vessels supports an increase in mitochondrial function in co-opted tumors and allows neoplastic growth without triggering angiogenesis. In angiogenic tumors, there were higher levels of expression of genes coding for membrane vesicles, angiogenesis, and remodeling- and inflammation-related pathways, possible due to more hypoxia. Supporting this finding, we observed a significant lack of fibrosis/desmoplasia and a reduction in inflammation in tumors with co-opted blood supply in comparison to angiogenic tumors. This study also found a differential expression of genes involved in the regulation of apoptosis. The pattern of expression observed suggests that more apoptosis occurs in angiogenic tumors, as would be expected from hypoxia. Hypoxia-inducible factor (HIF) target genes can also be induced by many oncogenes, so it will be of interest to investigate genetic changes in these cancers. In conclusion, the tumors with co-opted blood supply in this study have reduced inflammation, decreased apoptosis and efficient mitochondrial metabolism, deduced from gene expression array. However, new studies are needed to confirm these results.

## Primary and Metastatic Human Tumors

The capillary network of the brain parenchyma is one of the densest in the mammalian body and apparently a good basis for vessel co-option. An early indication that mechanism other than angiogenesis was important in tumor vascularization was based on results showing that the vascular density in human GBM was in the same range as that of normal cerebral white matter 5. Later, several comprehensive murine studies have added new knowledge to the impact of vessel co-option in brain tumors 30. Studies including patient samples have been rare, but in a recent study the impact of antiangiogenic treatment in patients with GBM was explored 31. Autopsy tissues from recurrent glioblastoma multiforme (rGBM) patients treated with pan-VEGF receptor tyrosine kinase inhibitor cediranib were compared to tissue from rGBM patients with standard care (surgery, radiation, and chemotherapy). The authors claim to provide the first morphological evidence that anti-VEGF treatment changes the GP of rGBM in patients with decreased microvascular proliferation, loss of pseudopalisading necrosis, and diffuse spread into the adjacent normal brain. Furthermore, they show that instead of switching to alternative angiogenesis pathways, rGBMs exhibit a more infiltrative phenotype and blood vessels with normal molecular expression and morphology after antiangiogenic therapy. However, the small number of autopsies and rGBM heterogeneity warrants further studies to confirm these findings.

As shown in Table 2, several studies have explored vessel co-option in NSCLC and the prevalence of tumors predominantly presenting a picture of vessel co-option reported to be about 10–20%. Interestingly, a subtype of NSCLC: bronchoalveolar carcinoma (BAC), has long been known to grow along alveolar walls preserving rather than destroying the original lung structure. Nonangiogenic lung tumors, however, typically entirely fill the alveoli and their histological subtype is clearly identifiable (e.g., squamous cell carcinoma or adenocarcinoma). In NSCLC the prognostic impact of a vessel co-option pattern has been addressed and although the results have been conflicting, several studies have found low degree of angiogenesis (a low endothelial cell proliferation fraction) to be associated with a poor prognosis 24.

Several studies have described nonangiogenic tumor growth in liver metastasis (Table 2), and in a recent study the histological GP of colorectal liver metastasis is also shown to have a prognostic value 8. The GPs were categorized according to the morphology of tumor liver parenchyma described in earlier similar studies: desmoplastic pattern, the tumor was separated from the liver parenchyma by a layer of desmoplastic stroma infiltrated with lymphocytes and nests of tumor cells; pushing pattern, the liver plates were compressed, running parallel to the tumor liver interface without desmoplastic stroma and only a mild inflammatory infiltrate; and replacement pattern, tumor cells and liver parenchyma were in close approximation with no compression of the plates, no desmoplastic stroma, or inflammatory infiltrate and the tumor cells replaced the hepatocytes in the liver cell plates without destruction of the liver architecture. Van den Eynden and coworkers conclude that the ones with a replacement GP (27.8%) are nonangiogenic, whereas the ones with a pushing GP (15.6%) are the most angiogenic with angiogenesis being, at least partially, hypoxia driven. At 2 years of follow-up, a GP with a pushing component was an independent predictor of poor survival, suggesting that the pushing GP is characterized by more aggressive tumor biology.

Another interesting question is whether metastases from angiogenic primary tumors share the vascular phenotype of their primary. Studies of the vasculature of metastases indicate that a switch to angiogenesis in the primary tumor is not a prerequisite for tumor progression to metastasis. Edel et al. 32 compared levels of angiogenesis in primary breast tumors and their matched lymph node metastases by studying endothelial cell proliferation and found no consistent association. Furthermore, Naresh et al. 10 investigated lymph node metastases from squamous cell carcinomas of the oral cavity and larynx which showed that metastatic tumors in lymph nodes have low MVD and low fractions of proliferating endothelial cells in comparison to the primary tumors. This indicates that angiogenesis may not be necessary for the growth of carcinoma metastases in the well-vascularized environment of the lymph node. This is borne out by the preferential location of metastatic cells in the highly vascular paracortex rather than in the follicles 10. In conclusion, tumor vascularization in a metastatic deposit is likely to be dependent on other factors besides the angiogenic capability of the clone from which it is derived.

## Clinical Implications

As discussed, tumors do co-opt host tissue vessels, it is not exceptional, and may be present in a large proportion of tumors. In the last decade research related to angiogenesis has been massive, but investigation related to vessel co-option as an alternative blood supply for tumor growth has been more limited.

An interesting question is whether the same pathways are activated in co-opted vessels as in angiogenesis. As previously mentioned, the major players so far known for helping endothelial cells to survive during co-option are VEGF and angiopoietins. Ang-1 activates Tie-2 and favors tumor vessel maintenance. However, upregulation of Ang-2 disturbs the interaction between Ang-1 and Tie-2 and causes destabilization of capillary walls. If the goal is to break down EC function one might think that a combination of an Ang-2 stimulator or Ang-1 inhibitor with a VEGF inhibitor may be an interesting approach. Targeting both angiopoietin/Tie2 and VEGF pathways is currently under investigation in phase I, II, and III studies 33, but the impact of vessel co-opted tumors has not been addressed. However, targeting angiopoietin/Tie2 pathway has been challenging as angiopoietins can exert either pro- or antitumorigenic effects, depending on the cellular context 33. In addition, it is observed that blocking VEGF signaling increases co-option and growth of satellite tumors 34. Furthermore, as previously mentioned, tissue from rGBM patients shows a picture consistent with increased vessel co-option after treated with pan-VEGF receptor tyrosine kinase inhibitor cediranib 31. Furthermore, in a murine GBM study, Lu et al. 35 observed inhibition of VEGF signaling leading to a proinvasive phenotype in a subset of GBM patients treated with bevacizumab. Although, whether this increased invasion may facilitate vessel co-option remains unanswered. Increased knowledge related to the impact of VEGF inhibitors on co-opted vessels is therefore warranted.

Interestingly there are, to our knowledge, no other growth factors associated with vessel co-option 36, but this is probably due to few studies related to this topic. We have, however, previously observed that tumors with co-opted blood supply had higher levels of genes coding for proteins involved in mitochondrial metabolism 29. Furthermore, tumors with co-opted blood supply had reduced inflammation and decreased apoptosis. These results have to be validated and further explored, but if there are such fundamental differences between tumors predominantly with angiogenesis versus co-optioned blood supply this may have major impact on targeted therapy strategies.

Another immediate implication relates to surgical resection of isolated secondary deposits. The phenomenon of isolated organ metastasis usually involves one of the three major organs discussed above: liver, lung, or brain, and can be seen with several different tumor types, for example, colorectal, breast, melanoma, and renal cancer. In itself, this suggests the possibility that rather than angiogenesis enhancing the colonization of multiple organs, a few metastases have been able to co-opt preexisting vessels. Overall, therefore, the tumor may not have such an aggressive phenotype. Patients who undergo liver resection for hepatic metastasis from colorectal cancer (CRC) experience recurrence rates ranging 60–85% 37. Predictive biomarkers, including angiogenic factors, have been investigated in this setting, but further well-designed studies are necessary to clarify their clinical relevance 37. It would be of interest to investigate whether there is an association between a vascular co-opted pattern and recurrence rate after liver resection for hepatic metastasis. For patients with colorectal liver metastasis, studies have shown that there are many tumors with an angiogenic phenotype: in contrast, the large majority of cases with breast cancer had a co-opted vascular pattern 7. Although only speculative, this may be one of several potential explanations why the VEGF inhibitor bevacizumab so far is proven more effective in metastatic CRC compared to advanced breast cancer.

Some studies have indicated that vessel co-option is typically located at the edge of tumors and, interestingly, less effect of antiangiogenic treatment in the tumor periphery is observed 38–41. In a recent murine study, using an experimental model of lung metastasis and the FDA-approved antiangiogenic drug sunitinib, Welti et al. 41 found in some tumors extensive central devascularization, but that the rim of these refractory tumors continued to be well vascularized. Vessel co-option may be one of several potential explanations for this observation. In general, vessel co-option is only one of many related mechanisms that may explain resistance to antiangiogenic treatment, as for instance, other modes of tumor vascularization (intussusception, vasculogenesis, and VM) 13, alternative proangiogenic factors 42, vascular maturation 43, activation of autophagy 44, or recruitment of myeloid-derived suppressor cells 45.

However, as co-opted vessels are also important in early stage of tumor development it would be of interest to know whether vessel co-option partly may be one of the reasons why antiangiogenic treatment has not succeeded so far in an adjuvant setting 46,47.

Prospective studies to address whether co-opted vessels are predictive for treatment response to antiangiogenic drugs are of interest. In addition to antiangiogenic-targeted therapy this also may be an issue in radiotherapy where tumor tissue's oxygen level is important for treatment effect. As discussed there are several methods to determine whether the vasculature is new or co-opted and this would be valuable in drug development, ensuring that appropriate patients were entered into clinical trials and trial resources were used more effectively. In addition to morphological characteristics and measuring endothelial proliferation, imaging methods such as DCE, MRI, or PET (positron emission tomography) probes for hypoxia will be important to monitor vessel co-option/angiogenesis status in tumors prior to and during treatment. Finally, more basic research on the underlying mechanisms with regard to vessel co-option is pivotal to develop potential new treatment strategies.

## Conclusions

Some tumors, both primary and metastatic, use preexisting host tissue vessels as their blood supply. In many cases, however, there is a mixed phenotype of co-opted vessels and angiogenesis. The fact that tumors may grow to a clinically detectable size without angiogenesis makes them less likely to respond to drugs designed to target the abnormal vasculature produced by angiogenesis. Even if only the invading edge of the tumor remains able to progress without angiogenesis, one may speculate that these drugs are likely to be ineffective. Despite massive research on antiangiogenic treatment, the understanding of vessel co-option is rather limited. There are indications that other biological mechanisms are important in tumors with co-opted blood supply than in angiogenic tumors, and further studies to explore the biological and clinical implication of these co-opted vessels are highly warranted.
